# Experimental Gait Analysis to Study Stress Distribution of the Human Foot

**DOI:** 10.1155/2017/3432074

**Published:** 2017-11-02

**Authors:** Vidya K. Nandikolla, Robin Bochen, Steven Meza, Allan Garcia

**Affiliations:** ^1^Department of Mechanical Engineering, College of Engineering and Computer Science, California State University Northridge, Northridge, CA 91330, USA; ^2^Department of Electrical and Computer Engineering, College of Engineering and Computer Science, California State University Northridge, Northridge, CA 91330, USA

## Abstract

Researchers and clinicians are increasingly using plantar pressure and force measurement system to evaluate foot functions. This research evaluates the quality and reliability of a Tekscan HR mat to study the plantar pressures and forces acting during walking, running, jumping, and standing of healthy subjects. The following regions of the foot were investigated: heel, mid foot, metatarsophalangeal joint, hallux, and the toes. The arches of both feet of the three healthy subjects in the gait analysis were presented which addresses the balancing issues of the body during locomotion. The results indicated that the peaks at the big toe (79.4 ± 8.5 N/cm^2^,* p* = 0.0001) were the maximum compared to forefoot (40.3 ± 3.3 N/cm^2^,* p* = 0.001), to midfoot (7.5 ± 1.3 N/cm^2^,* p* = 0.001), and to heel (27.8 ± 3.9 N/cm^2^,* p* = 0.0002) for jump activity. The running activity demonstrated similar results as jump where the maximum peak pressures were absorbed at the big toe region. The heel region during running (86.3 ± 12.6 N/cm^2^,* p* = 0.001) showed three times the pressure peak compared to the jump land (27.8 ± 3.9 N/cm^2^,* p* = 0.0002) activity. The measurement system proved to be highly capable of detecting heel strike and toe-off moments.

## 1. Introduction

In recent advances, gait analysis has become a widely used tool to provide kinematic and kinetic data required by the physical therapists and doctors for choosing suitable treatment for their patients. Since the 1960s, study of gait analysis became more famous in clinics compared to research labs, as gait measurements were found useful in the management of patients with walking disorders. For humans, walking is like a speech or a breathing activity. The study of human locomotion has been conducted for many decades that describe the relationship between the motion and the muscle. Foot motion study has been amplified and complemented by a continuous stream of technological advances over a century. Full understanding of a normal gait includes study of muscle activities during different phases of the gait cycle. Advances in the study of muscle activities were made available during the 1940s and the 1950s. The contributions in the study of mechanical analysis of gait cycle were available in the 1950s that performed free-body diagrams and calculations that developed the effects of hip, knee, and ankle joints for the ground reaction forces. Many research studies have also focused on the mathematical modeling to demonstrate the motion of the body segments and actions of different muscles since the 1960s. Great improvements came in the 1970s and the 1980s in gait measurement methods. This led to accurate kinematic study using electronics rather than images that took a long time to gather information. Force platforms and EMG systems were made available that produced reliable results in minutes. 

These innovations provided high quality three-dimensional (3D) data on the kinetics and kinematics of walking and created a sophisticated mathematical model that could calculate muscle, ligament, and joint contact forces during human locomotion [1–4].

With the advances in gait analysis technology, the major limitation is not the ability to produce high quality data but knowing how best to use the data to benefit the patients. Clinical studies investigate the pattern of walking using one of two ways: motion analysis or visual observations. The first method (motion analysis) is expensive, as it requires dedicated equipment for motion capture, highly accurate computer based sensors, and uses cumbersome equipment attached to the patients. This method produces the most accurate data but for short distance locomotion. The second method (visual observation) is not as expensive as the first method as it does not require any special equipment but consumes a lot of time to collect data for multiple visits. The results are not completely reliable and also difficult to compare accurately with multiple visit data [5–7].

A normal human walking or running can be defined as locomotion of the two legs alternating to provide both support and propulsion. In other words, foot is the final segment that provides support to the body during the gravitational and inertial loads. Since the body weight impacts the foot locomotion, it is important to study how the forces move from heel to mid stance to push off regions. Human gait is the study of locomotion where the legs swing forward. The function of the foot during gait analysis is widely used to evaluate magnitude of forces acting, timing of motor unit function, walking abnormalities for presurgical assessment, and treatment follow-ups. In order to understand an abnormal gait, it is necessary to study the normal gait and process the information. For example, a normal gait analysis can be compared only between similar age groups and similar body geometry types, meaning an appropriate standard needs to be chosen before the individual's data is studied. An elderly women gait data cannot be compared with a healthy young fit women data, as there will be larger differences, whereas comparisons with normal elderly women data may show the normal limits of her age and sex. Also, another important information that needs to be understood is, not all abnormal gait is in some ways undesirable, meaning many gait abnormalities are a compensation of some other problem and these abnormal gaits are nonetheless useful for those individuals to balance the foot motion [2, 4, 8]. Having said that, it is important to understand the terminology of the gait study and its relationship with the foot motion, which are described in the later sections in this paper.

## 2. Background

**Figure 1:**
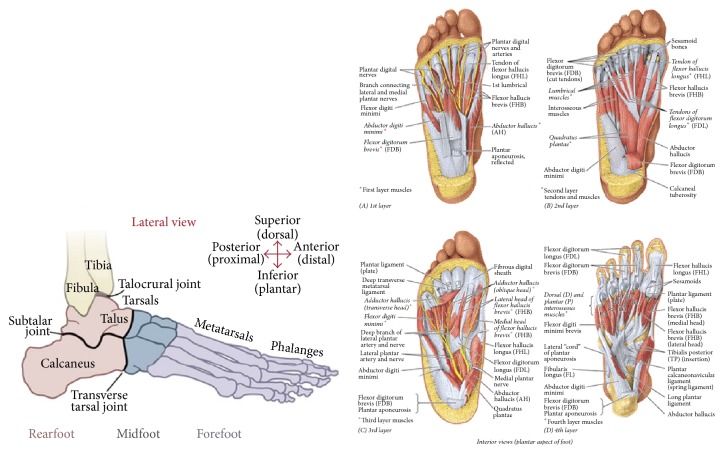
Bottom left: bones and major joints and regions; bottom right: third layer muscles; top right: first layer muscles.

The study of locomotion and kinematics of the human foot is a common practice of researchers investigating rehabilitation and human impairment treatment [9]. To better understand the locomotion of the foot, various models are used as a simplified representation. The majority of dynamic representations of the foot have been limited to two-dimensional models [10–12]. This simplification allowed the foot to be modeled as a rigid body, although the foot is a much more complicated structure with many individual muscle layers, bones, and joints that make up the system as seen in Figure 1. More recent models have been created that try to accurately represent the foot by separating the foot into a three-dimensional segmented model [13, 14]. This approach allows the segments to more accurately depict the motions of their counterpart. For instance, instead of the entire foot being modeled as a 2D rigid body, the talus and calcaneus bones can be modeled as two rigid body segments of several. Therefore, joints like the subtalar, which provide rotation between these two segments, can be incorporated in the model [14].

Although the advancements in modeling the foot continue to be made, they are still limited to our understanding of the locomotion and kinematics of the foot. There are many techniques used to study the kinematics of the foot including motion capture, surface marker, electromyography, force platforms, and pressure measuring systems [15–19]. The technique we intend to use is the pressure measuring system. Studies have shown that plantar pleasure measurement systems are an effective method for measuring impulse, pressure applied over time, or the area beneath a pressure time curve, as compared to standard force platform [15–17, 20]. The limitations to this method include its inability to measure the shear forces involved in dynamic motions. However, the pressure measurement system we intend to use has been shown to be a reliable tool for plantar force and pressure measurements during dynamic movement [20].

**Figure 2:**
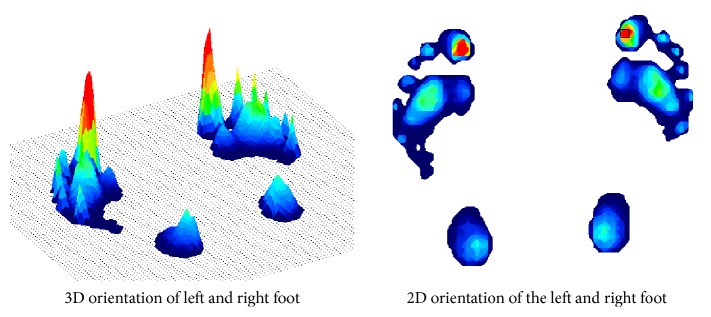
2D and 3D plot of planter pressure distribution.

For assessing the foot function, planter pressure study is been increasingly used. Pressure mats or force platforms are designed to analyze plantar pressures and forces during ambulatory activities.  Figure 2 shows an example of how a 2D and 3D plantar pressure distribution is explored. The peaks in the plot depict the high pressure regions, meaning high forces act in those areas during locomotion. The color-coding demonstrates the increase in pressure intensity from light blue to dark red color [21].

In this research study, Tekscan HR mat is used to record the data and measure the plantar pressure, force, and stress distribution during standing, walking, running, and jumping. The study of the stress distribution of the human body during locomotion is described in the later sections along with results and discussion.

## 3. Methods

The dynamic performance of the foot is studied for different activities. During walking or running, human foot exerts a force on the surface, which in turn exerts an equal and opposite force that is termed as ground reaction force. The ground reaction forces and plantar pressure measurements of the foot are used to study the stress concentration effects on the plantar surface. With the technological advances in the pressure sensing equipment, measuring the vertical component of the forces and the contact area at different locations of the foot has become commercially available for research as well as for clinical applications. Research studies have recognized that the elevated plantar pressures are a causative factor in the development of pedal pathologies, stress factures, plantar calluses, and neuropathic ulceration. Identifying proper treatment of such elevated plantar pressures can play an important role in management of lower limb disorders [25–29]. Therefore, the primary aim of this study is to present the experimental plantar force and pressure data during various activities using a high-resolution (Tekscan HR F-scan system) pressure mat.

### 3.1. Hardware/Sensors

The sensor mat used is the Tekscan HR F-scan system, with 8448 sensels on a surface area of 0.572 m by 0.457 m (0.261 m^2^). The mat is 0.18 mm thick. This comes to a sensel density of about 32 sensels per square cm or a sensel area of about 0.31 square cm per sensel. Each sensel works on a principle of piezoresistivity, which is the raw data that is read by the VC-1 VersaTek cuffs, converted to a digital signal with 256 increments (8 bit), and then sent via Cat-5e cables of 4 ft with Rj45 Connectors to the VersaTek Datalogger (VWD-1) and then via USB 2.0 (480 Mbps) to the lab PC [21]. 

**Figure 3:**
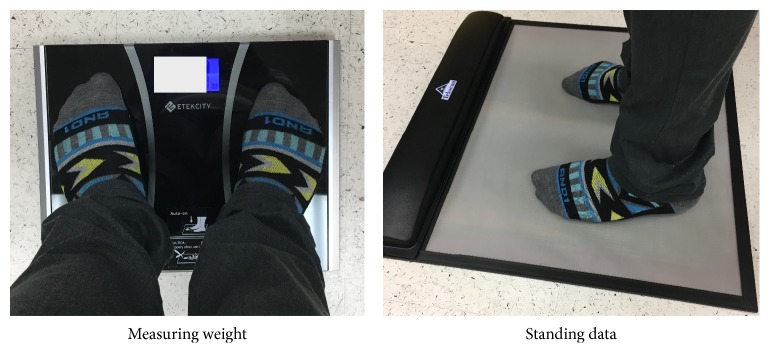
Etekcity weight scale (left) used to record the mass of the subject and standing position (right).

The HR F-scan sensor mat is calibrated for each user, which takes into account the user's weight. The weight is collected using a household high precision Etekcity Weight Scale EB9312H (as seen in Figure 3). The process of calibration involves the subject either to take a step or to walk. To maintain greater accuracy and precision in our research study, we used the step variation method to calibrate before collecting any data. This process involved stepping on the mat when prompted, with either foot and maintaining the balance for a minimum of 3 seconds. The calibration serves to correlate the 256 increments of raw data from each sensel to an absolute value in a desired unit; in our case, we used kilograms, and a direct change to pressure was available within the software.

The software used is HR Mat research 7.0 by Tekscan. For each set of data collected, a new movie is recorded. All of our recordings were done at 180 fps and maximum sensitivity, with the exception of the jump trials, which required a medium sensitivity to avoid saturation of the sensels during the user's landing. The videos started recording from the first frame, a force over 1 Kg is registered and then until it registers no forces at all.

### 3.2. Experimental Setup and Procedure

The data collection process consists of five parts for each subject: calibration, standing, gait, running, and jumping. Since only one set of equipment is available, each subject will go through all five parts successively. The applicants that meet the criteria will be asked to appear for testing at roughly 15-minute intervals to ensure efficient testing. All parts of the data acquisition are conducted with the subject in socks. Calibration is the first process, which requires the subject to be weighted before any data is collected. The value is averaged over three trials to check repeatability and is used as an input for the calibration. A step calibration setting was used on the Tekscan HR F-scan system. The initial position for the step calibration is the subject standing off the mat. The proctor then clicks a start button, and a timer appears in the calibration window. After one or two seconds, the computer directs the subject to rapidly step onto the mat and balance them self on one foot for three seconds to be properly calibrated. The step calibration is done before the trials of interest are recorded.

The standing test is the first of four trials of interest. For this recording, the sensitivity of the mat is set to “high” due to the low range of pressures. Higher sensitivity is required to ensure the most accurate baseline. The standing trial serves as the reference for the other trials. The subjects station themselves in the center of the mat (as seen in Figure 3), while standing up straight and looking forward. After holding the position for ten seconds, the proctor starts the data collection. Data is recorded for ten seconds to ensure a constant reading. The subject is then directed to step off the mat and back on. The process is repeated three times and the results are averaged. This data was recorded as a baseline for the pressures experienced during an inactive state.

**Figure 4:**
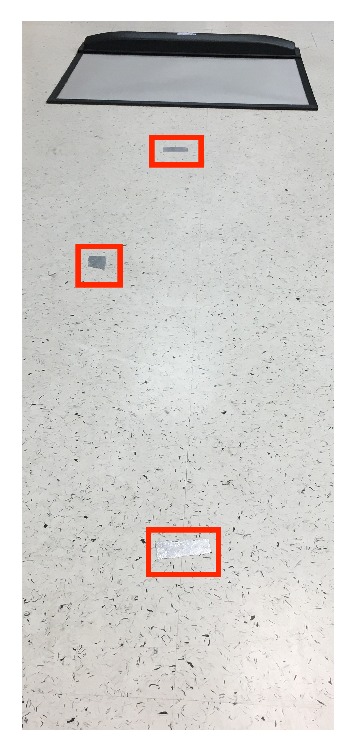
Image of a taped path for walking/running gait test.

For running trials, the sensitivity setting was changed to default. The sensitivity is lowered because there is a greater range of pressures. The first of the two tests conducted was the gait. To ensure repeatability between trials, the subject conducted multiple practice gait cycles. As the subject walked, their step was marked by tape (as seen in Figure 4). This created a path tailored to the subject's natural gait. A full gait cycle was placed in between the start point and the mat. This was done to record a sample of data that excluded an initial take off. As the subjects walked they were asked to look straight ahead and to only use their peripheral vision to follow the guide tape if needed. The process of the running test is the same as the gait. For the scope of this study, running is defined as a speed just above walking, where only one foot can be on the ground at any time.

For the jump trial, the sensitivity was reduced to the lowest setting. This setting was chosen because the largest ranges occur during the jump trial. A jump, in this study, is defined as a six-inch lift from a two-foot takeoff and landing. The six-inch lift was measured using six-foot six-inch reference pole. Black tape was used to mark the subject's eye level and six inches above that. The subject stood feet planted on the mat and eyes pointed straight ahead at the lower taped mark. They were prompted to take practice jumps to the height where their eye level was aligned with the higher tape mark. Once the subject was comfortable, the proctor prompted the subject when to jump for the data collection.

### 3.3. Human Subject Selection Criteria

Three (3) male subjects were selected for the initial study with an age range of 22 to 26 years of age. The applicants were asked to disclose any known conditions that effect the walking for the accuracy of the study. Before the experiment was conducted, the subjects were advised on the procedures and steps involved for the data collection process. The students were also advised to wear socks and follow the markers during the walking and jumping process.  Table 1 shows the mean height of 176 cm and mean weight of 87.5 kg.

Inclusion criteria were as follows:

- Subject must be over 18 years of age.
- Subject should be within a regular height range, approximately 5′2′′ to 6′2′′.
- Subject should be representative of an average healthy young adult.

Exclusion criteria were as follows:

- Subject has recently experienced any damage to bone or muscular structure that currently impacts their ability to walk (i.e., broken leg, rolled ankle, and pulled muscle).
- Subject may be considered grossly over obese or underweight (BMI under 18 or over 35).

User profile contains the following:

- Subject number (assigned)
- Height
- Weight
- Sex
- Age
- Ethnicity
- Frequency of physical activity
- Disclosure of any relevant medical conditions
- Does the subject use foot inserts

The described inclusion, exclusion, and user profiles are advertised for recruiting the subjects for the study. They are also a part of the survey that the subjects fill out to apply for the research study. The entire data collection process will follow the approved IRB protocol to maintain the safety of the data.

## 4. Results and Discussions

The data analyzed was the product of three iterations of the following tests: right foot walking, left foot walking, right foot running, left foot running, jumping, and standing. The tests with the left and right foot were separated by simply conducting a test focusing on either foot at a time. These tests were repeated for three case studies (CS_1, CS_2, and CS_3) with the participants being able to repeat iterations of the procedure if they thought that they were conducting the walk, run, or jump abnormally. All tests were analyzed using excel from data extracted from the HR Mat software.

The subjects were 24 ± 2.1 years of age. They weighed 85.3 ± 16.4 kg and had heights of 176.3 ± 8.3 cm. The three subjects were healthy at the time of the study, though one had a history of back issues. Foot types of the subjects: CS_1 has a normal arch, CS_2 has a high arch, and CS_3 has a flat foot.

### 4.1. Walking

**Figure 5:**
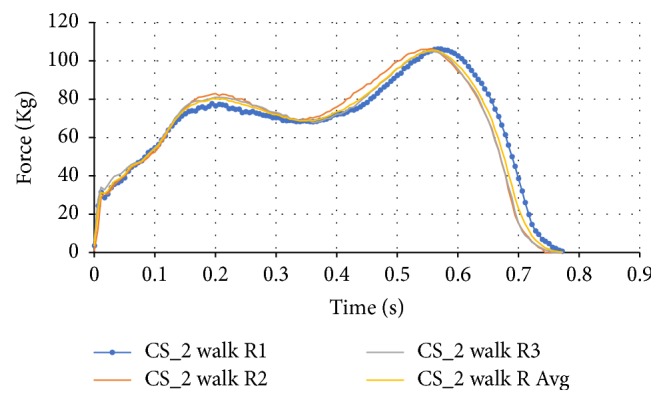
Walking data for CS_2's right foot.

For the walking tests the force data was averaged between the three given tests for both the left and right foot. The time of these tests was not averaged between the three results when graphing because this would cause either the omission or addition of forces at certain times. Though the time was not averaged, the averaging for the three walking tests was indicative of an average walking test as shown in Figure 5. The peak forces were slightly shifted to the more average time while remaining very close to the peak forces measured. This includes the heel strike portion at the beginning of the test.

**Figure 6:**
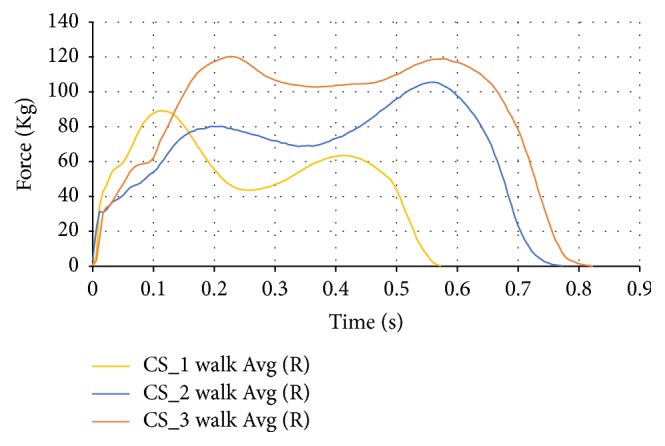
Walking comparison data for the right foot of the subjects.

The averages for the walking tests were compared between the subjects that are shown in Figure 6. Different subjects at different weights, heights, and arches produce very different walking profiles. For example, CS_1 has high initial peak forces with low ending peak forces, CS_2 has high ending peak forces with low initial peak forces, and CS_3 has similar peak forces during both periods. These differences in pressure peaks are also effected by the different foot types of the subjects.

**Figure 7:**
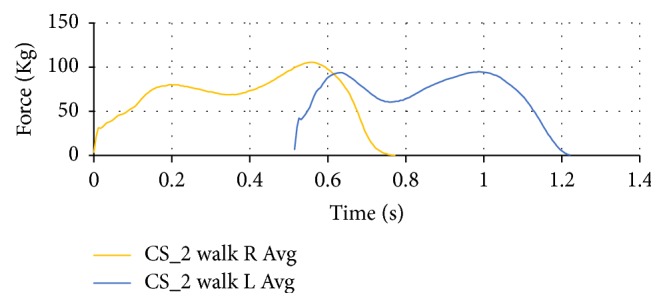
Plantar forces for right and left foot to approximate gait cycle of CS_2.

The right and left foot walking data are overlaid with a given offset so as to provide an approximate gait cycle as shown in Figure 7. This is an approximation as the first force (heel strike) of the opposite foot occurs at two-thirds through the stride of the other foot.

**Figure 8:**
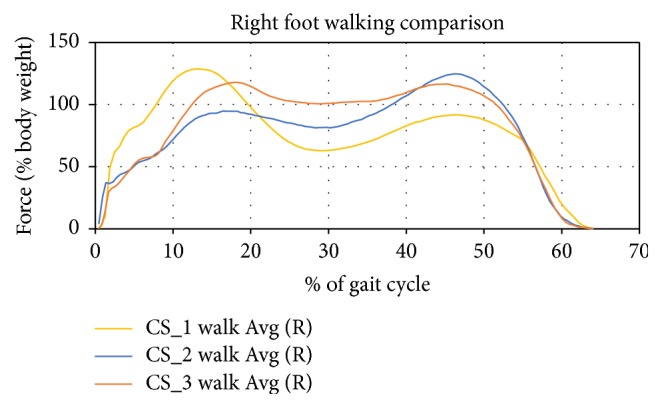
Forces in body weight% over gait cycle%.

**Figure 9:**
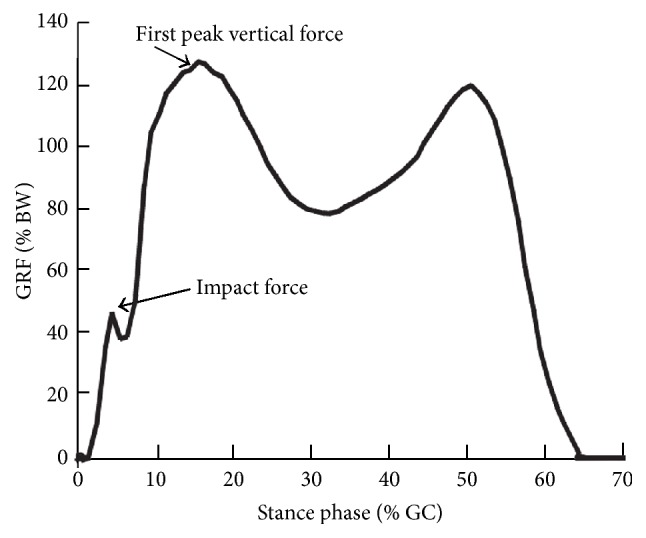
Forces in body weight% over gait cycle% [22].

The average data for the three case studies was normalized for body weight and the percentage of the gait cycle elapsed in Figure 8. This data was then compared to Figure 9 where ten subjects had the ground reaction force measure and normalized using a similar method and equipment [22].

**Figure 10:**
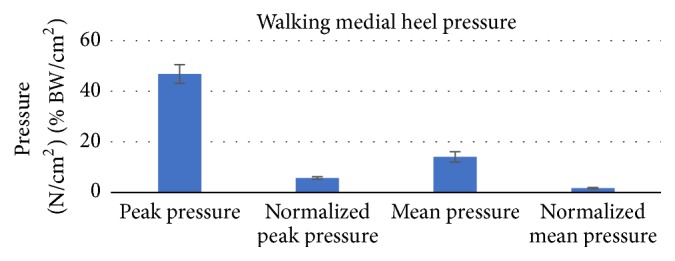
Walking medial heel peak and average pressures (absolute and normalized).

**Figure 11:**
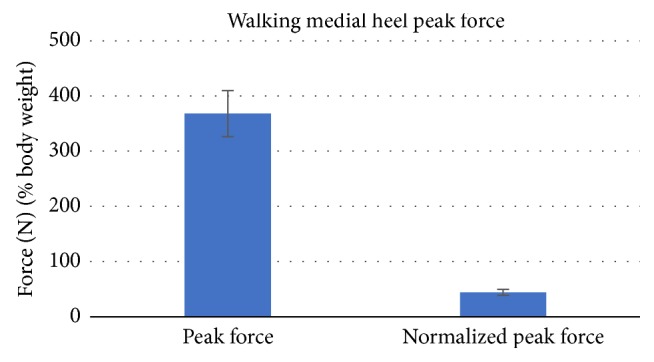
Walking medial heel peak forces (absolute and normalized).

**Figure 12:**
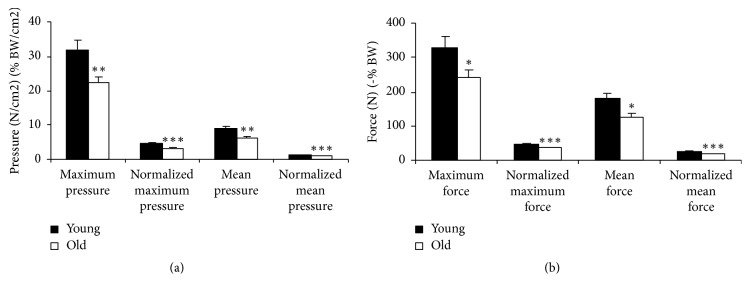
Walking medial pressure [^*∗∗*^maximum and mean:* p* = 0.01; ^*∗∗∗*^normalized maximum:* p* = 0.001; ^*∗∗∗*^normalized mean:* p* = 0.0006] (a) and force [^*∗*^maximum:* p* = 0.05; ^*∗*^mean:* p* = 0.02; ^*∗∗∗*^normalized mean:* p* = 0.0006; ^*∗∗∗*^normalized maximum:* p* = 0.001] (b) [23].

The walking data was also analyzed for the medial part of the heel as seen in Figures 10 and 11 and compared with other research studies as shown in Figure 12 [23]. Since the case studies were relatively young, only the young portion of Figure 12 should be considered.

### 4.2. Running

**Figure 13:**
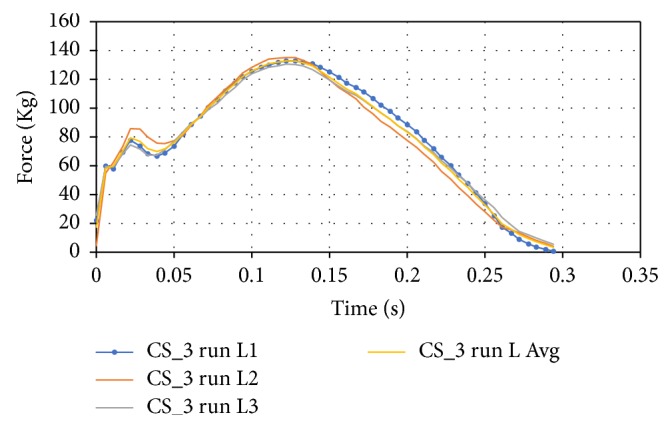
Running data for the left foot of CS_3.

Similar to the walking test, the running test separated the values for the right foot and the left foot; however, the gait cycle is not shown as it would require times between the right foot toe off and the left foot heel strike. The forces are once again averaged between the three trials for the left foot as shown in Figure 13.

**Figure 14:**
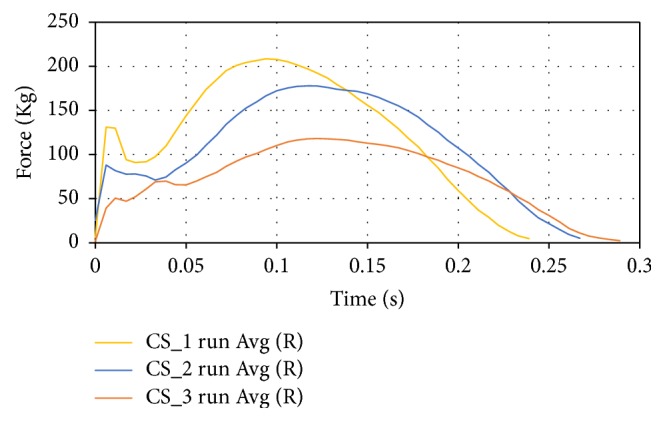
Comparison of the averages for the right foot gait of the subjects.

The running test averages were also compared for the three case studies as shown in Figure 14.

**Figure 15:**
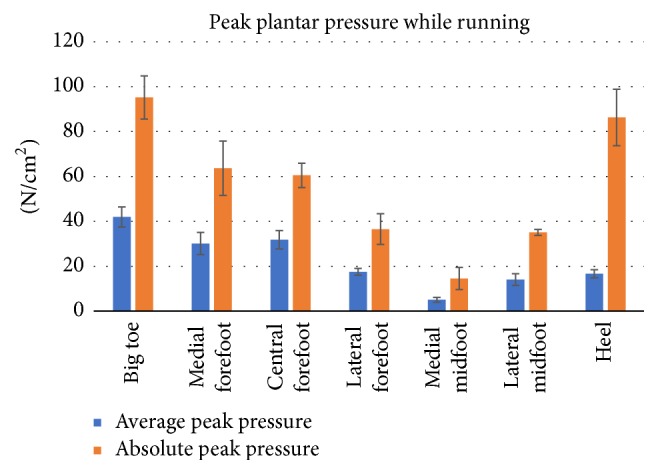
Segmented plantar pressures while running without shoes.

**Figure 16:**
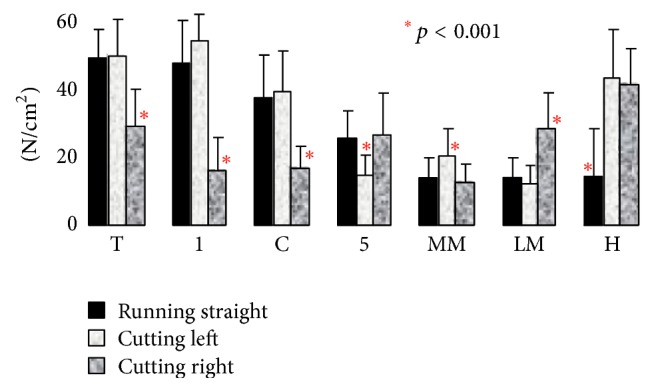
Segmented plantar pressure while running with shoes [24].

Data from the running trials were segmented in Figure 15 to represent different parts of the foot and compared with other research studies shown in Figure 16 [22]. Because the case studies did not have shoes, a good representation of the data was the average peak pressures in Figure 15 (*p* < 0.0001), which has similar representation with shoes on as seen in Figure 16. Since no changes in direction were captured, the “Running Straight” portion of Figure 16 should be focused. For Figures 15 and 16, big toe is represented by (T), medial forefoot (1), central forefoot (C), lateral forefoot (5), medial midfoot (MM), lateral midfoot (LM), and the heel by (H).

### 4.3. Jumping

**Figure 17:**
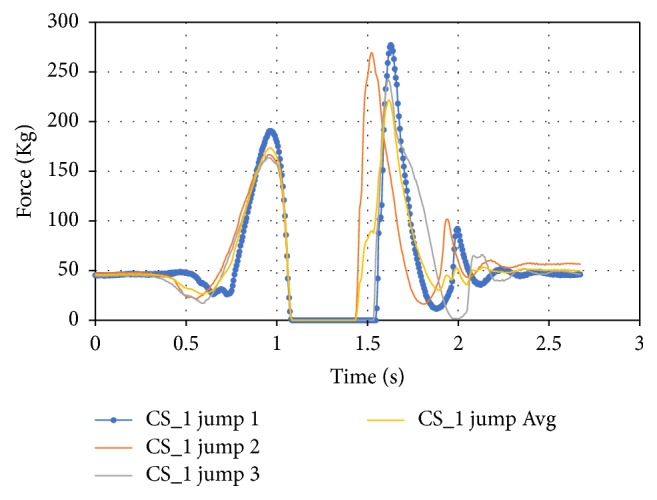
Overall jump average for CS_1.

**Figure 18:**
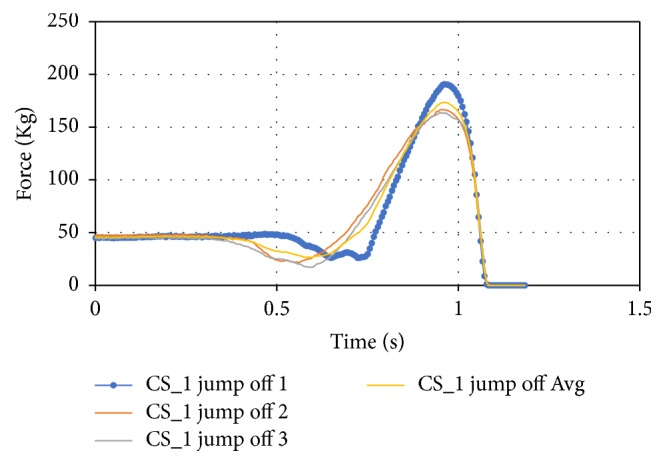
Jump off average for CS_1.

**Figure 19:**
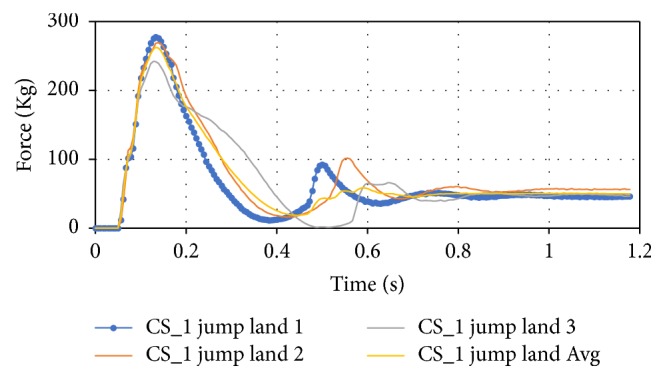
Jump land average for CS_1.

For the jumping, three tests were performed for each subject. Both the left and right foot forces were captured during the same time. Although they were compared and averaged, there were slight differences between the timing of the jump off to the jump land. To improve the results of the jump data it was separated into three sections: the overall jump average of the case study, the jump off average, and the jump land average that are shown in Figures 17, 18, and 19.

**Figure 20:**
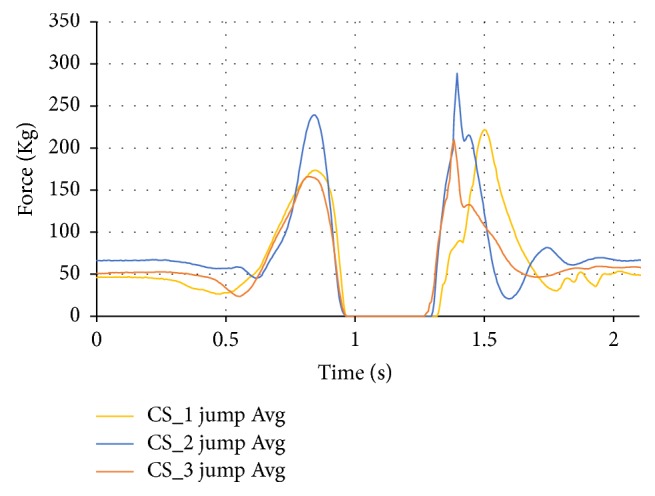
Jump comparison data between the subjects.

Another downside to averaging the jump data is that the spring action of the foot while landing is averaged so it does not accurately show this action. This data can be separated individually and analyzed more in the future. The data between the jump, jump off, and jump land averages were also compared for the three case studies. The overall jump average can be seen in Figure 20 where CS_2's jump average was indicative of an average jump.

**Figure 21:**
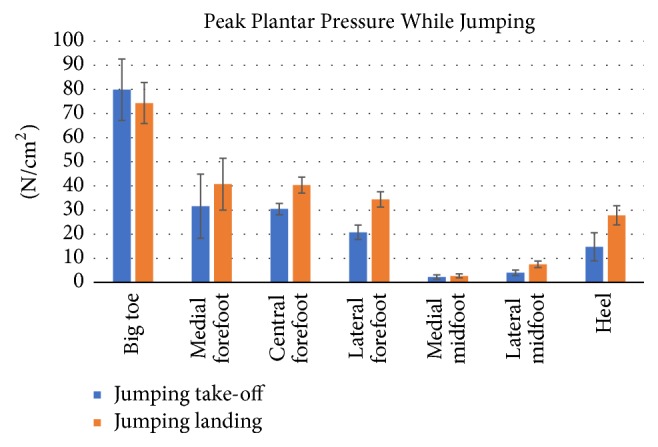
Peak plantar pressures while jumping and landing.

**Figure 22:**
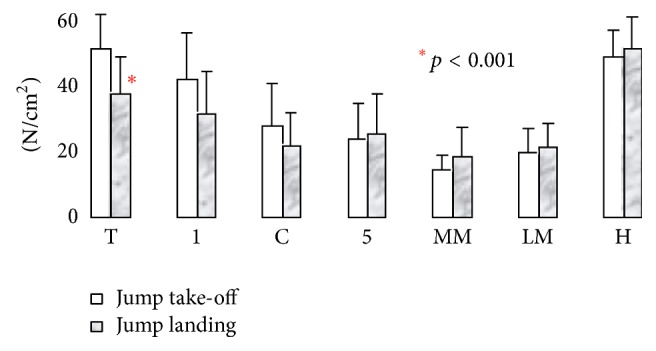
Peak plantar pressures while jumping and landing [24].

The jump data was segmented similarly to the running data for comparison to jump data from other research studies [22, 24]. Although it followed similar trends (such as low medial and lateral midfoot forces), the two data sets had drastically different peak forces as seen in Figures 21 and 22. This can be attributed to the lack of shoes worn by the case studies' during trials. Without the cushion from a shoe, peak forces are unable to spread. The large standard deviation in the trials of Figure 21 can be attributed to a case study behaving as an outlier.

### 4.4. Standing

**Figure 23:**
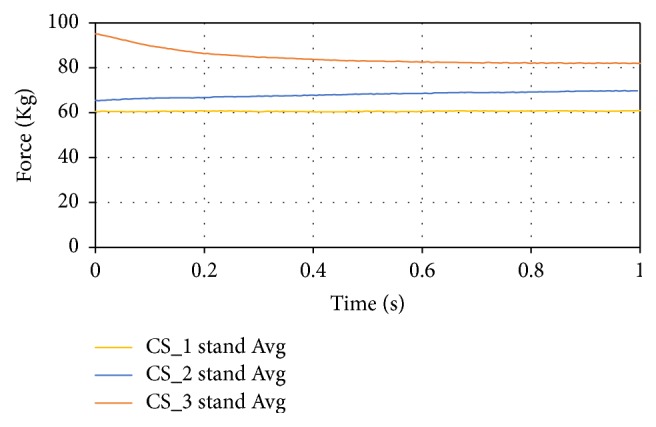
Average standing data of the subjects.

The standing data was averaged between three tests for each subject. The averaged standing data for the subjects are shown in Figure 23. Over time, the data sets reached an equilibrium force for the subjects within a ten percent error of their actual weights.

### 4.5. Plantar Force and Pressure Analysis

Peak pressure analysis helps identify and quantify peak plantar pressure area. It displays force and pressure curves over time, demonstrates frame-by-frame single and multiple stances, and displays center of force pathway and its trajectory.

**Figure 24:**
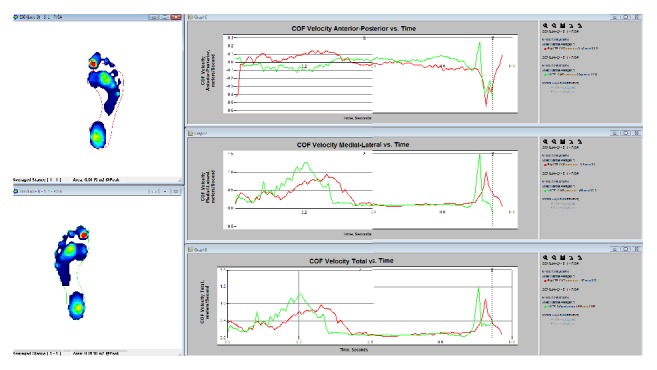
Velocity analysis for left (green) and right (red) foot during walking.

Figure 24 illustrates the movement of the center of force (CoF) during the gait cycle. The top graph shows the velocity of the CoF from anterior to posterior (back to front), the middle graph shows the velocity in respect to the medial line of the foot (side to side), and the last graph shows the total velocity of the CoF.

**Figure 25:**
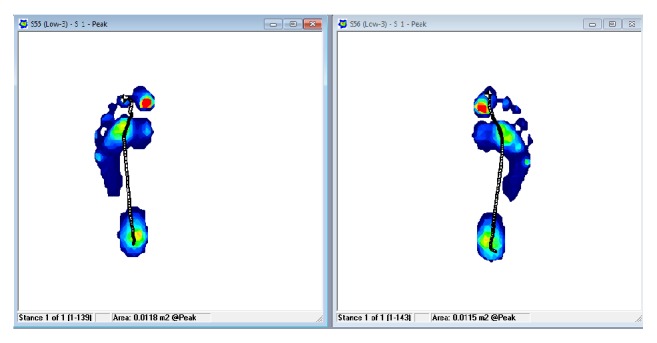
Center of gravity pathway for left and right foot during walking.

**Figure 26:**
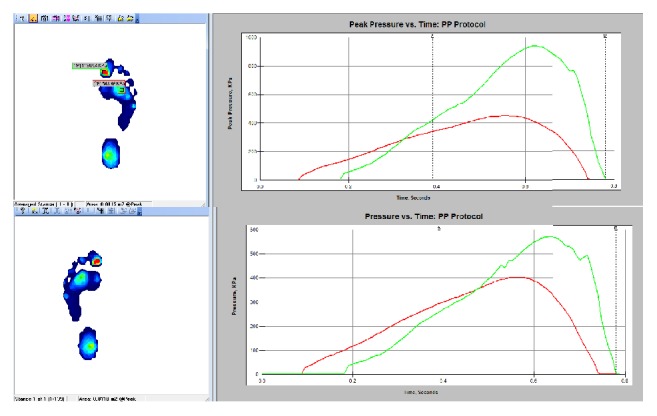
Peak pressure analysis for left and right foot.

During walking, the CoF travels to support the weight of the body for balancing and propelling forward.  Figure 25 shows the CoF pathway for both feet which are stitched into one graph for display.  Figure 26 shows the pressure peak during walking.

The peak pressure analysis shown in Figure 26 is an option given by the software to find and analyze the points of peak pressure during the gait. As the center of gravity path moves as seen in Figure 25, Figure 26 demonstrates the pressure distribution of both feet during walking. The green and red lines represent the pressure peak points with respect to time.

**Figure 27:**
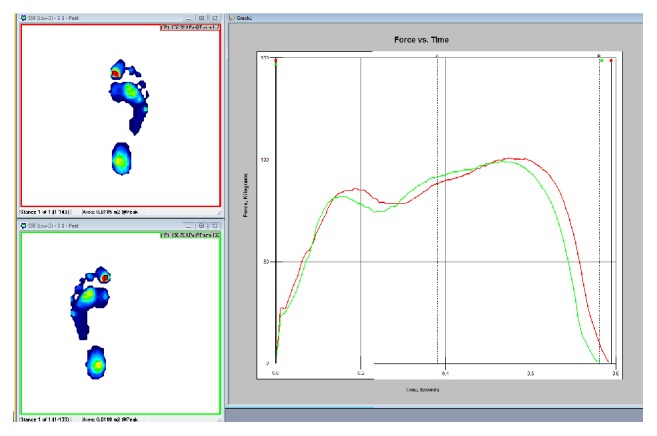
Plantar force versus time for gait analysis (left (green) and right (red) foot).

**Figure 28:**
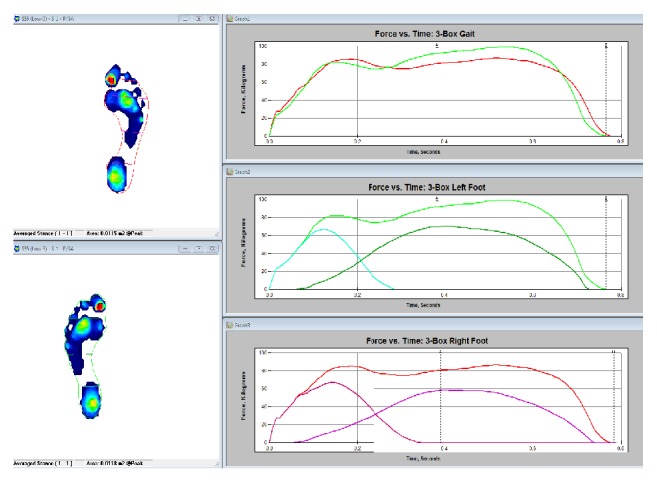
3-box plot left and right foot.

**Figure 29:**
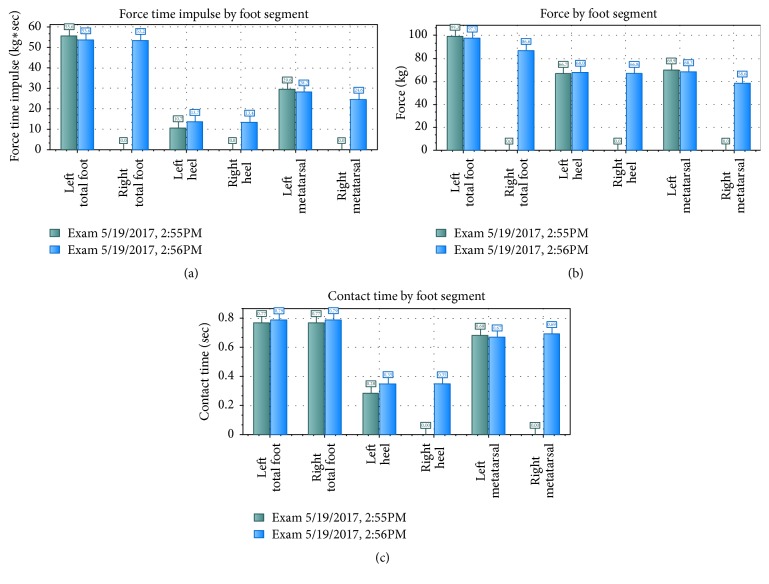
Foot function and 3-box gait report.

The gait force versus time plot shown in Figure 27 shows the force distribution throughout the gate cycle for both feet. The plot shows the characteristic curve associated with a single pendular motion. When the heel strikes the ground, the force rises sharply. As the weight of the person is distributed on the heel, the force stops growing and begins to fall as the weight is distributed to the entire foot. The pressure then grows as the weight shifts forward and spikes as the weight is focused on the mid stance and toe. As the heel and toe lift, the force drops again. The 3-box analysis shown in Figures 28 and 29 provides a quick visual of pressure and force profiles of the feet. Foot function parameter includes contact times, loading and off-loading rates, center of force (CoF), velocities, and maximum force.

### 4.6. Statistical Analysis

The data values from Figures 10 and 11 are represented in Table 2 with mean ± standard deviation and *p* value. In the table, the walking medial peak force (N) represents the maximum force for a specific instance of time over the entire medial section of the heel. The experimental walking average data was compared with other research studies: 368.1 ± 41.7 N (*p* = 0.062) versus 328.4 ± 138.7 N (*p* = 0.049) [23]. The research studies data are presented in Figure 12. The peak pressure represents the maximum pressure at a small area in the medial heel section which was compared with other research studies (46.9  +/− 3.7 N/cm^2^ (*p* < 0.01) versus 32.9  +/− 11.8 N/cm^2^ (*p* = 0.01)) along with the mean pressure over the duration of step at the medial section of heel (14.1  +/− 0.4 N/cm^2^ (*p* = 0.04) versus 8.9  +/− 3.1 N/cm^2^ (*p* < 0.01)) [23].

The mean ± standard deviation and its *p* values for Figures 15 and 21 are represented in Table 3 for jumping and running cases. The peak plantar pressure represents the maximum pressure experienced in specific locations in the segmented region. The absolute peak data represents the maximum pressure at a specific point in the segmented area, while the average peak force represents the maximum pressure at a point in time over the entire area of the segmented section. The data as seen in Table 3 demonstrates the movement loads of anatomical regions of the foot during activities. Also, the data sets from the subjects indicate that the test procedure is viable for varying subject profiles (*p* < 0.002). However, as seen in Table 3, for some data, there was strong indication that the procedure failed to be repeatable (*p* > 0.002). Specifically during the running test in the lateral forefoot region, one subject did not make contact with the sensor mat, skewing the significance factor to *p* = 0.49 for the peak value. Such misinformation can be avoided by increasing the number of trials.

Specific maneuvers loaded the plantar surface of the foot in unique ways, which are summarized in Table 3 during running and jumping. Statistical data of the pressures during specific movements for the big toe (*p* < 0.001) to central forefoot (*p* < 0.001) to midfoot (*p* < 0.02) to heel (*p* < 0.03) were significant. For jump, the peak pressures for heel (N/cm^2^) for jump off and jump land are 14.8 ± 5.9 (*p* = 0.036) and 27.8 ± 3.9 (*p* = 0.0002) respectively, where, as for the big toe, the pressures (N/cm^2^) for jump off and jump land are 79.8 ± 12.7 (*p* = 0.001) and 79.4 ± 8.5 (*p* = 0.0001), respectively. The big toe region absorbs the maximum pressures during jump off and land activity. For running, the peak pressure (N/cm^2^) at big toe is 95.2 ± 9.6 (*p* = 0.00003) and heel is 86.3 ± 12.6 (*p* = 0.001), meaning both the regions are high pressure peak areas. The lowest pressure peak is at the medial midfoot region for running (14.5 ± 4.9 (*p* = 0.004)) as well as for jumping (2.7 ± 0.8 (*p* = 0.0002)). The highest pressure peak is at the big toe region for running (95.2 ± 9.6 (*p* = 0.00003)) as well as for jumping (79.4 ± 8.5 (*p* = 0.0001)). From the data, it is observed that the big toe and heel absorb the maximum pressure during the running activity in comparison to the jump.

## 5. Conclusion and Future Work

The healthy subject's data are shown in the result section along with comparison with other research studies using similar equipment. Although some approximation with respect to timing of the data was integrated (such as the stance phase taking 64% of the gait cycle), the data was averaged in a way that kept the vital forces accurate. The only situation where this was not the case was for the heel strike of the walking, running, and jumping data. For the heel strike for the jumping and running data, instead of having a sharp point that is expected, the heel strike is generally smoothed out over the first part of the data. However, this error is not large and can be accounted by finding the specific point in time where heel strike occurs for the three tests and averaging the data. The jump data was split into two different portions: the jump off and the jump land. This minimizes error due to the different times spent in the air. The spring action of the foot was not averaged properly with respect to the jump land data, but this can be remedied by using a similar solution to the heel strike data where specific points of data are chosen before they are averaged.

Information elicited from the figures in result section shows the analysis of the plantar pressure and force distribution for the study of dynamic foot function. The data was repeated to maintain the accuracy of the forces acting during different activities. Figures 24, 27, and 28 depict the information on how the pressure is distributed, the period of time, and how effectively the weight is being transferred between right and left foot. Overall, compared with current research studies, the data for walking, running, and jumping from our study was fairly consistent with that found in other articles [16–19, 22–24]. Also, having a larger amount of case studies can increase the accuracy of this study.

The information presented in this research paper is real-time data, similar to what is produced by sophisticated F-scan system that are used in podiatrist clinics. As foot is the weight bearing mechanism for human body, this dynamic assessment allows us to understand the kinematic motion during walking/running. The data represents the pressure and gait parameter analysis of the foot plantar pressure during daily activities and documents the gait speed, time with heel in contact with ground, which can be used, by the podiatrist or clinicians to depict the human gait disorder [30–32]. There are studies that link gait characteristics to gait deficiencies; for example, first symptoms of neurological disease is poor balance, slower pace, and gait support issues. These conditions are asymptomatic and may not be noticed for years until detected. Therefore, evaluation of gait may be valuable for early detection before the disease is prolonged [20, 32–34]. The results presented in this paper show the foot patterns, the arches of both feet during standing, walking, running, and jumping. The study displays the pressure-force patterns from heel to first metatarsal heel. The primary aim of the study is to determine the asymmetry between the right and left foot. The right leg acceleration pattern should have the similar pattern to the left. If the arches and pattern differ significantly between the left and the right foot, it means they have different joint flexion angles and joint position trajectories. These possible differences can do early detection of underlying problems.

The future research is to focus on wireless sensor mat accessing plantar pressure distribution for different types of foot inserts for aggressive activities [35, 36] like playing, dancing, and so forth. The phase II research utilizes the reliability of this data to compare and develop a smart foot insert with wireless communication that can be helpful for diabetic patients with neuropathy condition.

## Figures and Tables

**Table 1:** Subject profile.

Subjects	Gender	Age (yrs)	Height (cm)	Weight (kg)	BMI	Status	Medical history
Case Study 1	Male	22	167	69.2	24.8	Desirable	Healthy
Case Study 2	Male	23	183	84.6	25.3	Overweight	Healthy
Case Study 3	Male	26	179.07	108.86	34.0	Obese	Healthy

Average	—	24	176	87.5	34	—	—

**Table 2:** Statistical data for walking.

	Mean ± SD	*p* value
Walking medial heel peak force		
Peak force	368.1 ± 41.7	0.062
Normalized peak force	44.3 ± 5.5	0.062
Walking medial heel pressure		
Peak pressure	46.9 ± 3.7	0.00685
Normalized peak pressure	5.73 ± 0.5	0.00685
Mean pressure	14.1 ± 2.1	0.04
Normalized mean pressure	1.7 ± 0.3	0.04

**Table 3:** Peak plantar pressures (N/cm^2^) at regional locations for jumping and walking.

	Mean ± SD	*p* value
Jump off		
Big toe	79.8 ± 12.7	0.001
Medial forefoot	31.6 ± 13.3	0.22
Central forefoot	30.4 ± 2.3	0.00001
Lateral forefoot	20.8 ± 2.9	0.0001
Medial midfoot	2.2 ± 0.9	0.02
Lateral midfoot	4.0 ± 1.2	0.01
Heel	14.8 ± 5.9	0.036
Jump land		
Big toe	79.4 ± 8.5	0.0001
Medial forefoot	40.7 ± 10.7	0.01
Central forefoot	40.3 ± 3.3	0.001
Lateral forefoot	34.4 ± 3.2	0.00001
Medial midfoot	2.7 ± 0.8	0.0002
Lateral midfoot	7.5 ± 1.3	0.001
Heel	27.8 ± 3.9	0.0002
Running average peak		
Big toe	42.0 ± 4.5	0.00001
Medial forefoot	30.1 ± 5.0	0.0002
Central forefoot	31.8 ± 4.1	0.001
Lateral forefoot	17.5 ± 1.5	0.49
Medial midfoot	5.0 ± 1.1	0.0003
Lateral midfoot	14.0 ± 2.6	0.002
Heel	16.6 ± 1.8	0.001
Running absolute peak		
Big toe	95.2 ± 9.6	0.00003
Medial forefoot	63.6 ± 12.1	0.001
Central forefoot	60.5 ± 5.4	0.0002
Lateral forefoot	36.5 ± 6.8	0.44
Medial midfoot	14.5 ± 4.9	0.004
Lateral midfoot	35.0 ± 1.3	0.000001
Heel	86.3 ± 12.6	0.001
